# Exome profiling of primary, metastatic and recurrent ovarian carcinomas in a *BRCA1*-positive patient

**DOI:** 10.1186/1471-2407-13-146

**Published:** 2013-03-22

**Authors:** Jian Zhang, Yuhao Shi, Emilie Lalonde, Lili Li, Luca Cavallone, Alex Ferenczy, Walter H Gotlieb, William D Foulkes, Jacek Majewski

**Affiliations:** 1 Department of Human Genetics, McGill University, Montreal, QC, Canada; 2 Genome Quebec Innovation Centre, Montreal, QC, Canada; 3 Program in Cancer Genetcs, Departments of Oncology and Human Genetics, McGill University, Montreal, QC, Canada; 4 Departments of Pathology, McGill University and Jewish General Hospital, 546 Pine Avenue West, Montreal, QC H2W 1S6, Canada; 5 Departments of Obstetrics & Gynecology and Oncology, McGill University, Montreal, QC, Canada; 6 Lady Davis Institute and Segal Cancer Centre, Jewish General Hospital, Montreal, QC, Canada

**Keywords:** Driver mutations, Gynecological cancer, Hereditary cancer, Next generation sequencing, Tumor suppressor genes, Chromosomal rearrangements

## Abstract

**Background:**

Ovarian carcinoma is a common, and often deadly, gynecological cancer. Mutations in *BRCA1* and *BRCA2* genes are present in at least a fifth of patients. Uncovering other genes that become mutated subsequent to *BRCA1/BRCA2* inactivation during cancer development will be helpful for more effective treatments.

**Methods:**

We performed exome sequencing on the blood, primary tumor, omental metastasis and recurrence following therapy with carboplatin and paclitaxel*,* from a patient carrying a *BRCA1* S1841R mutation.

**Results:**

We observed loss of heterozygosity in the *BRCA1* mutation in the primary and subsequent tumors, and somatic mutations in the *TP53* and *NF1* genes were identified, suggesting their role along with *BRCA1* driving the tumor development. Notably, we show that exome sequencing is effective in detecting large chromosomal rearrangements such as deletions and amplifications in cancer. We found that a large deletion was present in the three tumors in the regions containing *BRCA1*, *TP53*, and *NF1* mutations, and an amplification in the regions containing *MYC*. We did not observe the emergence of any new mutations among tumors from diagnosis to relapse after chemotherapy, suggesting that mutations already present in the primary tumor contributed to metastases and chemotherapy resistance.

**Conclusions:**

Our findings suggest that exome sequencing of matched samples from one patient is a powerful method of detecting somatic mutations and prioritizing their potential role in the development of the disease.

## Background

Ovarian carcinoma (OC) is the leading cause of death from gynecological cancer in western countries. The most important predisposing factors are germline mutations in inherited cancer susceptibility genes, most notably *BRCA1*, *BRCA2*, *RAD51C, RAD51D* and the mismatch repair genes [1,2]. Recently, next generation (exome) sequencing of 316 OC revealed that over 20 percent of these cancers carried either somatic or germline inactivating mutations in either *BRCA1* or *BRCA2*, thus emphasizing the importance of these two genes in the pathogenesis of OC [3]. Notably, about a quarter of women diagnosed with OC in their fifth decade will carry a *BRCA1* or *BRCA2* mutation [4]. Several studies have observed that *BRCA1* and *BRCA2* mutation carriers tend to have a better outcome than stage-matched non-carriers, and that this better outcome is largely attributable to the combination of *BRCA* mutation status and DNA-damaging chemotherapeutic drugs such as cisplatinum [5]. There have also been case reports of rare cures achieved in *BRCA1/2* carriers with ovarian and other cancers following other, older treatments such as melphalan [6]. Together, these findings suggest that optimal alignment of chemotherapeutic agents with both host and tumor genetic events is possible and is in fact required to achieve improved outcomes. To further understand the interaction between treatment, host genetics and tumor-specific mutations, we extracted DNA from four sources obtained from a single patient carrying a deleterious mutation in *BRCA1* (blood, primary tumor, omental metastasis and relapse (recurrence) following standard post-operative therapy with carboplatin and paclitaxel). These four DNA samples were then subjected to whole exome sequencing, thus allowing us to identify tumor-specific variants and to determine potential changes in allele frequencies and emergence of new variants in the different tumor samples.

## Methods

### Clinical history

**Figure 1:**
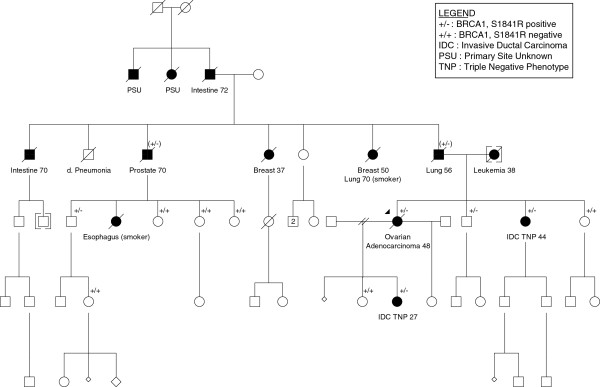
**Pedigree of the proband.** The person whose germ-line and tumor DNA was sequenced is indicated with an arrowhead (ovarian adenocarcinoma, age 48). Clear evidence of segregation between the mutation and breast and ovarian cancer is seen by the presence of triple-negative *BRCA1*-related breast cancer in her sister and daughter, who both carry the S1841R allele. Other carriers are indicated, with untested obligate carriers indicated as (+/−).

**Figure 2:**
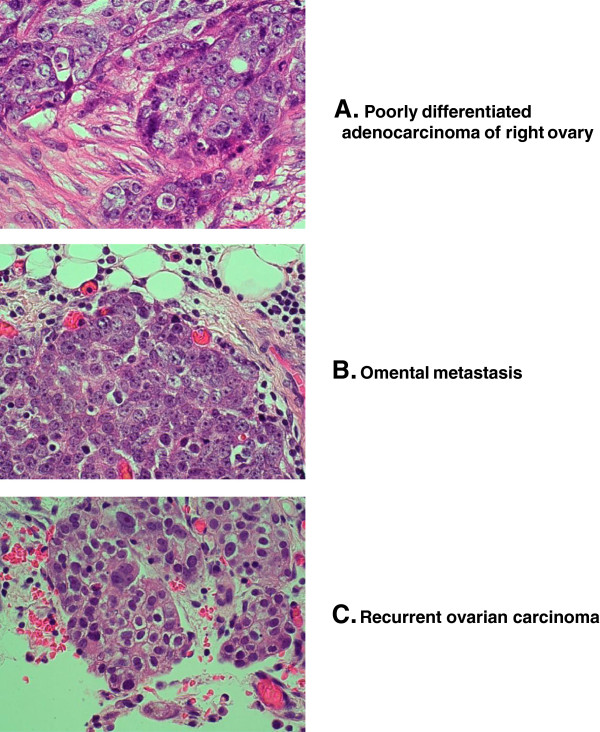
**Photomicrographs.** Representative frozen tissue was collected at the time of surgery, sections were stained with hematoxylin and eosin and DNA was extracted from the frozen tumors. Because the frozen sections were quite thick, they have not photographed well. We present here images of the paraffin-embedded tumors that reflect the frozen sections that were used for DNA extraction. The poorly differentiated original tumor appeared to be arising from the right ovary; **A** - solid proliferation of highly atypical epithelial cells with enlarged, pleomorphic nuclei and macronucleoli. H&E X400; metastases were widespread, and a biopsy was taken from the omentum; **B** - solid sheet of malignant cells displaying the same microscopic features as the primary ovarian carcinoma. The tumor cells invade the adjacent fibrofatty tissue of the omentum. H&E X400. Despite only minimal residual disease being present at the end of the primary surgical resection, the tumor clinically recurred after only three months of chemotherapy (discussed above) and at laparotomy, tumor was found on the surfaces of pelvic and abdominal organs and was biopsied: **C** - the malignant cells are smaller than the primary ovarian and omental carcinoma cells. They have clear, cytoplasmic and smudgy nuclear substance, and occasional giant macronuclei and nucleoli. These features may be a reflection of degenerative effects of previous chemotherapy. H&E X400.

The subject of this study was a 48 year old patient who had undergone total abdominal hysterectomy for menorraghia and left salpingectomy for ectopic pregnancy in the past. She had a family history of breast cancer (Figure 1), and was taken to the operating room in September 2003 by general surgery for a suspected diverticular abscess. She was found to have diffuse abdominal carcinomatosis with multiple masses throughout the abdominal cavity. Final pathology revealed a stage IIIc poorly differentiated serous ovarian cancer (Figure 2). Following three courses of neo-adjuvant chemotherapy with carboplatin (AUC = 6) and paclitaxel (175 mg/m^2^), her CA-125 dropped from a >3000 to 128 iu/l. She underwent optimal secondary interval cytoreduction with no residual disease. Samples were taken at this time (Figure 2). She was referred to the medical genetics service and a deleterious missense *BRCA1* mutation, c.5521A>C, S1841R, situated in the highly conserved BRCT domain of BRCA1 [7] was identified and found to be segregating with breast and ovarian cancer in her family (Figure 1). Despite further chemotherapy including adjuvant carboplatin-paclitaxel, paclitaxel consolidation, and cisplatin with gemcitabine, liposomal doxorubicin, topotecan, and thalidomide (all of which resulted in short-lived partial responses), the patient died of recurrent disease in August 2007. DNA extracted from the blood used for clinical *BRCA1* testing was subjected to exome sequencing. This study is approved by the Jewish General Hospital Research Ethics Office, Montreal, Quebec, Canada (Assurance Number 0796). Written informed consent for participation in the study was obtained from all participants.

### Tumor samples used for exome sequencing

Tumor samples were kept at −80 degrees Celsius. All examined tumor blocks contained poorly differentiated serous adenocarcinoma (Figure 2). The histiotype was ascertained in routine histological slides obtained from the same tumor which was fixed in formalin and sections were obtained from paraffin-embedded tissue. This was done because cell morphology was not preserved well enough to provide information on the histiotype of the malignant cells. The serous histiotype was further demonstrated by immunohistochemistry: the neoplastic cells of all tumor samples stained strongly and diffusely for CA-125, p16, TP53, Ki-67 and WTI. They failed to stain for caldesmon, fascin and only very weakly and focally for B-cadherin. This immunohistochemical profile is consistent with serous differentiation.

### Exome sequencing and SNP/small indel detection

Exome sequencing was applied on the primary tumor, the omental metastasis, the tumor present at relapse, and the blood from the patient to identify somatic mutations. Exomes were captured from a total of 3 μg of genomic DNA, using the Illumina TruSeq exome enrichment kit, according to manufacturer’s protocols. Samples were sequenced using one lane of paired-end, 100 bp reads on Illumina Hiseq for each sample. We ensured that only read pairs with both mates present were subsequently used. Adaptor sequences and quality trimmed reads were removed using Fastx toolkit ( http://hannonlab.cshl.edu/fastx_toolkit/). Reads that passed quality control were aligned to the UCSC hg19 reference genome with BWA [8]. Duplicate reads were marked using Picard ( http://picard.sourceforge.net/) and were excluded from downstream analyses. SAMtools was used to call SNV and indel variants [9]. Next, we applied additional quality control measures to all identified raw variants based on the following criteria: 1) The Phred-like score is no less than 20 for SNPs and 50 for indels; 2) the read coverage of no less than three reads per base; 3) at least three and 10% of covering reads had to support the alternate base for the primary tumor sample. Finally, we used Annovar to identify SNVs and indels that located in protein coding regions as well as variants affecting canonical splice sites [10].

We further filtered the variants against dbSNP and 1000 genome project data set, as well as previously identified variants by our lab from >100 exome sequencing blood samples unrelated to cancer. Only variants that have not been previously observed in any of the control exomes were considered potentially functional and selected for downstream analysis. The allele frequency of the variants was calculated as reads of alternate base/total reads. Variants with increased allele frequency from the primary tumor to the metastasis and the recurrence were selected for validation by Sanger sequencing. The PeakPicker software was applied to quantitatively measure the allele proportion of selected SNVs [11]. The allele proportion was calculated by:

$$\mathrm{Allele}\;\mathrm{proportion}=\;\frac{\mathrm{peak}\;\mathrm{height}\;\mathrm{of}\;\mathrm{alternated}\;\mathrm{base}}{\mathrm{peak}\;\mathrm{height}\;\mathrm{of}\;\mathrm{reference}\;\mathrm{base}}$$

To compare the allele frequency from exome sequencing and the allele proportion from Sanger sequencing, we converted the Sanger sequencing allele proportion to allele frequency as:

$$\mathrm{Mutant}\;\mathrm{allele}\;\mathrm{frequency}=\;\frac{1}{1+\frac{1}{\mathrm{allele}\;\mathrm{proportion}}}$$

### Copy number variant detection

Copy number variant (CNV) detection was done by comparing normalized read coverage or read-depth between the blood and each of the primary, metastatic, and recurrent tumors, using an algorithm based on ExomeCNV [12]. Read-depth was normalized to Reads Per Kilobase of exon model per Million mapped reads (RPKM) [13] for each exon, and the log ratio of RPKM values $\left(\log2\frac{\mathrm{RPK}M_{\mathrm{tumor}}}{\mathrm{RPK}M_{\mathrm{blood}}}\right)$ were calculated. Log ratios serve as input for DNAcopy, which segments chromosomal regions based on similar log ratios [14]. In this study, because the use of exome sequencing data is still not well proven in CNV detection, we refrained from attempting to identify small structural variants and concentrated on larger segments, which we can detect with high confidence. In order to identify large scale rearrangements, the DNAcopy outputs were smoothed by removing small CNV calls and merging adjacent segments. Some large CNVs may be represented by more than one segment because they span regions where exonic data are unavailable.

If there is no actual change in copy number between blood and tumor (the null hypothesis), then the ratio of RPKM values between blood and tumor should follow some distribution centered on 1. In fact, it follows a standard normal distribution after Geary-Hinkley Transformation (Let t be the transformed random variable). Therefore using t as a test statistic for each exon, a p-value can be calculated that gives the probability, under the null hypothesis, of finding a particular RPKM ratio as extreme as the one being observed. A smaller p-value means that it is unlikely to observe the given RPKM ratio under the null hypothesis, i.e. this gives an indication of copy number alteration at that exon. Let Ф(*t*)be the cumulative probability distribution of the transformed variable t, which follows the standard Gaussian distribution, then p for each exon is calculated as follows:

$$p=\left\{\begin{array}{c} 2\left(1-\Phi \left(t\right)\right)\;t\geq 1\\ 2\Phi \left(t\;\right)\;t<1 \end{array}\right)$$

In our present analysis, the identified regions contain at least 100 exons which collectively show deviation from the expected. The probability that all of these show the same deviation by random chance is negligible (i.e. if p-values for each exon within the segments are combined using Fisher’s Method, the resulting p-value approaches zero).

## Results and discussion

We obtained ~100 million sequencing reads that passed quality control for each sample. The mean read coverage in the blood, the primary tumor, the omental metastasis, and the recurrence was 174X, 130X, 162X and 146X per base, respectively, allowing for confident detection of mutations across the entire frequency spectrum. We searched for *de novo* somatic mutations by excluding all variants present in the blood from the list of variants detected in the three tumor samples (Table 1). Based on the criteria described in the Methods section, we identified 39 somatic mutations in the primary tumor and a greater number of somatic mutations in the metastasis and recurrence (47 and 52 mutations). However, we found that all of the primary tumor/metastasis/recurrence-specific mutations were identified from poor alignments or variant callings, and on visual inspection of the data, the remaining mutations were also detected in the primary tumor with small numbers of supporting reads.

**Table 1:** Numbers of variant calls from exome sequencing results

**Sample name**	**Raw variants**	**Variants after quality check**	**Rare variants after filtering**	**Somatic variants**	**Validated somatic variants**
OV	463944	200059	90	39	24/26
OMN	514227	230935	106	47	24/26
REC	487007	222994	95	52	24/26

OV= primary tumor; OMN=metastatic tumor; REC=recurrent tumor after chemotherapy.

We proceeded to examine the change in frequency of the *BRCA1* missense mutation (chr17, 41197766, S1841R) and observed increasing allele frequencies of this mutation: 0.48 in the blood, 0.57 in the primary tumor, 0.76 in the metastasis, and 0.72 in the recurrence. Upon validation using Sanger sequencing, this mutation showed consistent increase in frequency: 0.39 in the blood, 0.50 in the primary tumor, 0.68 in the metastasis, and 0.78 in recurrence. We note that the measurements from exome appear more accurate than from Sanger sequencing, because the allele frequency from exome sequencing of the inherited *BRCA1* mutation in the blood sample was closer to the expected 0.5, representing heterozygosity. Although we observed increase in frequency of this mutation from blood to tumor samples, we did not observe complete loss of the wild-type allele in the tumors. Based on previous investigations of series of *BRCA1* mutation-positive patients [3] the primary, metastatic and recurrent tumors will frequently exhibit complete loss of heterozygosity (LOH), and therefore the mutant allele frequency in the tumors should be close to 1, instead of 0.57 - 0.76, suggesting that the tumor samples may contain considerable proportion of non-malignant tissue. Allowing for sampling issues, it does appear that the frozen primary tissue (equivalent paraffin section images shown in Figure 2A) contains a considerable amount of non-malignant tissue, whereas, as shown in Figure 2B, the percentage of malignant tissue in the omental biopsy is higher (fat cells take up some of the sample, top of the figure). This is even more evident in Figure 2C, where there appears to be very little non-malignant tissue present. Further corroborating these data, CNV detection results showed that the allelic frequency of all the identified large deletions/duplications is increased from primary tumor to metastatic and recurrent tumors. Concurrently, we find no evidence for *de novo* alleles in the primary tumor that are absent in the subsequent tumors – which would have indicated that the primary tumor contained a mixture of different malignant clones. Thus, we hypothesize that the primary tumor sample we obtained for sequencing contained a relatively larger proportion of normal tissue than the metastases. The increased mutant allele frequencies among tumor samples are likely to reflect a more pure tumor sample, rather than a selection process. Moreover, CNV detection suggested that the region (17q11-17q21) containing *BRCA1* gene was deleted in all tumors, including the primary. This result is consistent with LOH, and that in this patient, the inherited mutation and the somatic deletion in *BRCA1* together initiated the tumor growth.

**Figure 3:**
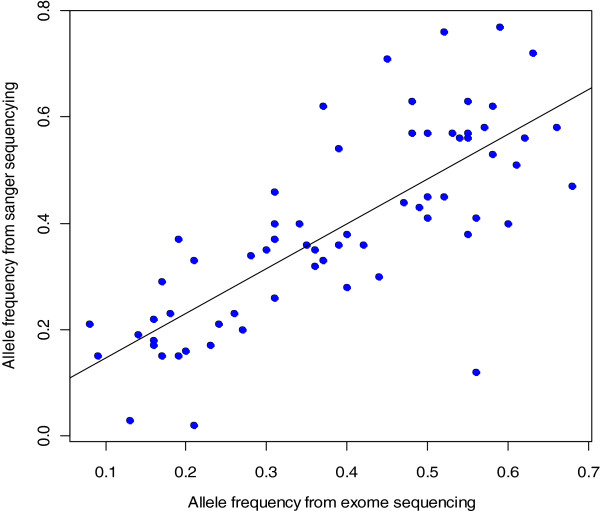
**Mutation frequencies by two different sequencing methods.** The correlation of mutant allele frequencies from exome sequencing and Sanger sequencing on validated mutations in the primary tumor, the omental metastasis, and the recurrent tumor after chemotherapy (Spearman’s rank correlation= 0.78, p = 7.865e-15).

In order to validate the exome sequencing results, and further investigate the possibility of selection of driver mutations during the evolution of the tumor, we selected 26 variants with supporting reads increased by at least 10% in the metastatic or post-therapy tumors. Sanger re-sequencing validated 24/26 mutations as being present in all three tumor samples but not in the blood sample (Table 1). We found high concordance of the allele frequency estimates from exome and Sanger sequencing (R = 0.78, p = 7.865e-15, Figure 3). The degree of concordance between the two methods renders high confidence in the selected candidate gene list. However, as mentioned above, we believe that the increase in allele frequency of most of the mutations is a result of difference in tumor purity, as opposed to a selection process.

The above observation implies that most of the detected mutations were present in the primary tumor and that very little, if any selection has occurred thereafter. The most compelling hypothesis regarding the origin of *BRCA1*-related high-grade serous ovarian carcinoma is that in fact the majority of them arise in the fallopian tube [15]. Our findings suggest that most of the critical tumor-driving clonal evolution occurs very early in the life of *BRCA1*-related highgrade serous carcinomas. One can reasonably speculate that the three tumors we studied here were all in fact “secondary” to the primary origin of the tumor and metastases from the now obscured primary tumor, likely in the fallopian tube. Surgery and chemotherapy failed to eradicate the original clone. Furthermore, when taking into account the relatively lower purity of the primary tumor, it is highly likely that most of the somatic mutations detected in this study were already present at high allelic frequency and high level of clonality in the tumor arising in the ovary. In agreement with our data, Castellarin et al. have recently suggested that in high-grad serous carcinoma patients, most somatic mutations found in recurrent tumors during platinum-based chemotherapy were present in primary tumors [16]. Our data thus suggests that little genetic evolution of the tumor has taken place from time of diagnosis to relapse following three courses of highly-active chemotherapy. It is possible that the 2.5 fold increase in allele frequency of the *NF1* mutation from the primary tumor to the metastasis (Table 1) indicates that this mutation appeared in the primary tumor later than for example, *TP53* mutation but was required for the full metastatic phenotype. It is likely that the primary tumor that is detected in patients is descended from cells that already contain a significant and potentially lethal mutational load.

**Figure 4:**
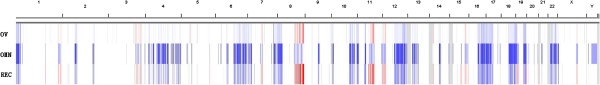
**Copy number variants in the ovarian tumors.** Filtered CNVs in the OV, OMN, and REC tumors across the genome, with chromosomal labels at the top. Because we are only interested in large scale deletions and amplifications, smaller CNV calls were removed and adjacent segments were merged. In the heat map, red indicates amplifications and blue indicates deletions. The magnified CNV patterns from OV, to OMN, to REC are likely due to differences in tumor purity. Notable amplifications are seen in 8q and 11q. Deletions are seen in chr4, 6q, 7q, 12q, 16q, chr17, chr19, chr22.

Another notable feature of our results is the presence of important cancer-related mutations (Table 1, Figure 4) and their corresponding structural rearrangements in all three tumors. Clear examples are the above-mentioned *BRCA1* mutation, the missense mutation in *TP53* resulting in R110P, the mutation in *NF1* damaging the donor site for splicing, and the deletion in region 17q11-17q21 which removed one copy of each of these three genes. In the recent companion study of ovarian carcinoma, *TP53* mutations were present in the primary, first recurrent and second recurrent tumors in three high-grade serous carcinoma patients [16]. Frequent somatic mutations in *NF1* have been previously shown to co-occur with *TP53* mutations [17]. The *NF1-*associated RAS pathway is also activated in many ovarian cancer cases [3,17]. Novel mutations identified in other genes (Table 1) should also be considered as candidates for intensive investigation, since they were identified from all three samples. An interesting candidate mutation is the D891N change in *TARBP1* (Polyphen score 1.00) [18]. *TARBP1* encodes an RNA binding protein with a methyltransferase domain. Methyltransferases have previously been shown to be involved in cancer [19]. Two somatic mutations (A1198A, W893*) in this gene have recently been found in ovarian cancer [3]. Our results suggest that in the primary tumor, *BRCA1* mutations might, in combination with *TP53*, *NF1* and *TARBP1* mutations contribute to the metastasis and relapse after chemotherapy. Analyzing the interaction between the RAS, BRCA1 and TP53-mediated pathways in ovarian cancer could be therapeutically worthwhile, especially if considered in combination [20,21].

We also show that valuable additional information regarding structural rearrangements can be derived from exome data. The CNV landscapes in our samples are associated with known ovarian cancer mutations (Tables 2, 3). Interesting examples include the amplification of 8q, which is likely driven by the MYC oncogene, and the amplification of 11q13, which is common in breast and ovarian carcinoma [22]. In addition, we observed deletion of chromosome 4, which has been shown to house several tumor suppressor genes, and deletions in chromosome 4 are associated with BRCA related tumours [23]. These mutations are likely acting combinatorially to drive the development of ovarian cancer. It is interesting to note that all of these genomic rearrangements are already present in the primary tumor, suggesting that large scale mutations accumulate quickly in early oncogenesis of ovarian cancer.

**Table 2:** Sanger sequencing confirmed somatic mutations with increased frequencies in tumor samples

**Position**	**Gene name**	**Mutation type**	**Mutant allele frequency from exome sequencing**			**cDNA change**	**Protein change**	**Polyphen score**	**Mutant allele frequency from sanger sequencing**		
			**OV**	**OMN**	**REC**				**OV**	**OMN**	**REC**
chr10:106124579	*CCDC147*	nonsynonymous SNV	0.31	0.45	0.52	c.G529T	p.A177S	0.29	0.40	0.71	0.76
chr17:38173081	*CSF3*	nonsynonymous SNV	0.26	0.49	0.66	c.C493T	p.P162S	0.61	0.23	0.43	0.58
chr15:64496758	*CSNK1G1*	nonsynonymous SNV	0.31	0.50	0.48	c.C881G	p.R294T	1.00	0.46	0.57	0.57
chr17:11696980	*DNAH9*	nonsynonymous SNV	0.24	0.42	0.62	c.A8222C	p.D2741A	0.12	0.21	0.36	0.56
chr4:88533803	*DSPP*	nonsynonymous SNV	0.27	0.61	0.52	c.T465A	p.N155K	0.96	0.20	0.51	0.45
chr20:33874597	*FAM83C*	nonsynonymous SNV	0.16	0.44	0.40	c.G1985A	p.T662M	0.00	0.17	0.30	0.38
chr6:5369392	*FARS2*	nonsynonymous SNV	0.2	0.36	0.35	c.G589A	p.V197M	1.00	0.16	0.35	0.36
chr14:25076412	*GZMH*	nonsynonymous SNV	0.17	0.40	0.37	c.G540T	p.Y180X	NA	0.15	0.28	0.33
chr10:126477647	*METTL10*	nonsynonymous SNV	0.14	0.57	0.60	c.T256C	p.I86V	0.06	0.19	0.58	0.40
chrX:153040228	*PLXNB3*	nonsynonymous SNV	0.17	0.21	0.19	c.G3898C	p.G1323R	0.06	0.29	0.33	0.37
chr12:3692299	*PRMT8*	nonsynonymous SNV	0.30	0.55	0.55	c.G904A	p.D302N	1.00	0.35	0.57	0.58
chr2:65316194	*RAB1A*	nonsynonymous SNV	0.18	0.37	0.39	c.T299C	p.N100S	0.00	0.23	0.62	0.54
chr7:122338859	*RNF133*	nonsynonymous SNV	0.17	0.36	0.34	c.C114T	p.W38X	NA	0.15	0.32	0.40
chrX:30870990	*TAB3*	nonsynonymous SNV	0.09	0.37	0.39	c.C1615T	p.E539K	0.07	0.15	0.33	0.36
chr1:234565362	*TARBP1*	nonsynonymous SNV	0.28	0.50	0.53	c.C2671T	p.D891N	1.00	0.34	0.45	0.57
chr17:7579358	*TP53*	nonsynonymous SNV	0.21	0.47	0.68	c.C329G	p.R110P	0.85	0.02	0.44	0.47
chr7:158824649	*VIPR2*	nonsynonymous SNV	0.13	0.63	0.59	c.G1081T	p.L361M	1.00	0.03	0.72	0.77
chr16:72828578	*ZFHX3*	nonsynonymous SNV	0.23	0.54	0.58	c.C8003T	p.R1754Q	0.45	0.17	0.56	0.53
chr19:58420819	*ZNF417*	nonsynonymous SNV	0.19	0.56	0.5	c.G827C	p.S276C	0.89	0.15	0.41	0.42
chr17:29554310	*NF1*	splice site SNV	0.16	0.56	0.48	c.G2325+1A	NA	NA	0.18	0.12	0.63
chr19:46192605	*SNRPD2*	splice site SNV	0.31	0.58	0.55	c.G3-781A	NA	NA	0.26	0.62	0.63
chr3:195022735-195022753	*ACAP2*	frameshift deletion	0. 15	0.41	0.55	c.1267_1285del	p.R423Wfs*26	NA	NA	NA	NA
chr1:201983017-201983030	*ELF3*	frameshift deletion	0.17	0.15	0.34	c.866_879del	p.N289Kfs*7	NA	NA	NA	NA
chr13:108922263-108922263	*TNFSF13B*	frameshift deletion	0.17	0.36	0.31	c.20delG	p.E8Sfs*15	NA	0.21	0.22	0.37

OV= primary tumor; OMN=metastatic tumor; REC=recurrent tumor after chemotherapy.

**Table 3:** Copy number variants (CNVs) that were detected in primary, metastatic and recurrent tumors

**Region**	**Type**	**CNV segements indicating deletion/amplification**					
		**OV**		**OMN**		**REC**	
		**Coordinates**	**Mean log ratio**	**Coordinates**	**Mean log ratio**	**Coordinates**	**Mean log ratio**
1p35-1p36	Del	861393-12980233	−0.3979	861322-27589726	−0.5557	861322-27589726	−0.5263
		13910301-22895846	−0.391	28059114-29652173	−0.5209	28059114-29650008	−0.5321
Chr4	Del	264888-42088143	−0.1326	264888-1389640	−0.5261	264888-1389640	−0.5903
		42145445-88235112	−0.1643	20255439-145040934	−0.5052	18023221-141832508	−0.5217
		88258428-190874280	−0.1689	148785997-189026086	−0.5084	147227078-190873442	−0.5302
6q16-6q25	Del	153313992-170176161	−0.2665	96971022-170893669	−0.5104	96969750-170893669	−0.5462
8p21-8p23	Del	117024-28385681	−0.287	190896-28385681	−0.5488	190896-28385681	−0.5817
8q21-8q24	Amp	90775210-122641580	0.5658	90926305-95709154	0.5043	91836945-97172920	0.5658
		123963751-142226069	0.98	97605708-122641580	0.927	97243283-121357802	0.9853
		142227189-145278133	0.5909	123963751-145725582	1.3829	121379410-145622144	1.4429
		145515440-146279543	0.5688				
11q12-11q14	Amp	64676463p-134251918	0.1758	63581159-94354158	0.7324	63766427-94354158	0.7829
12p12-12p13	Amp	250451-6637339	0.1653	247439-22089608	0.4673	247439-22089608	0.4963
		6638679-9262631	0.188				
		9264755-13140266	0.3317				
		13208485-31107009	0.2592				
12q21-12q24	Del	31116761-121883221	−0.1361	65078567-113909303	−0.5148	64668681-133781116	−0.5465
		121970711-131616361	−0.3135	114282473-133781116	−0.55		
		132195775-133781116	−0.3871				
16q21-16q24	Del	3725325-90142318*	−0.2189	50102691-90030718	−0.5425	50069328-69988476	−0.563
						70428885-90142318	−0.5792
17p + 17q11-17q21	Del	171206-7755654	−0.3947	63643-36881851	−0.5335	63643-36709091	−0.5552
		7758393-18286499	−0.3397	36894606-41234592	−0.5191	36865426-41256973	−0.546
		18539775-42328956	−0.3036				
19p13.3	Del	374421-8429523	−0.448	474621-8194249	−0.5189	110679-8402712	−0.5409
19p13.2	Amp	8555110-11531615	0.1418	8429206-18541740	0.4018	8429206-10625687	0.4414
		11559037-16639066	0.1043			10677734-11031424	0.8088
						11031510-18548570	0.4299
19q13.2-19q13.4	Del	17317922-59082756	−0.2849	41626252-59082756	−0.5468	41306478-59082756	−0.5686
22q	Del	17073440-18909917	−0.362	16448824-51133476	−0.517	17071767-51065188	−0.5632
		19029320-42999166	−0.3716				
		43023310-51065480	−0.4172				

OV= primary tumor; OMN=metastatic tumor; REC=recurrent tumor after chemotherapy.

## Conclusions

This work used whole exome capture and massively parallel DNA sequencing to study targeted candidate mutations in selected genes, as well as performing a “hypothesis-free” analysis where we aimed to identify potential driver mutations by identifying variants with increased proportion of mutant alleles. Genetic evolution of tumors from diagnosis to relapse after chemotherapy was not observed. Instead, we suggest that most of the critical tumor-driving and chemotherapy resistant mutations were already present in the primary tumor. We show that high-throughput sequencing is effective in detecting large chromosomal rearrangements such as deletions and amplifications that occur in cancer. It is notable that the patient responded very poorly to platinum-based therapy; relapse after only 3 course of therapy usually betokens a very poor survival. This early platinum failure is somewhat less common in *BRCA1*-related cancer than in non-hereditary ovarian cancer [5], and it seems unlikely that this failure is related to type of mutation (i.e. missense mutation) that was present in this patient. The large number of deleterious somatic mutations present in the primary tumor likely contributed to the rapid progression of the disease. It will be important to conduct studies such as ours in large numbers of patients to establish whether specific exomic profiles at initial diagnosis are associated with subsequent resistance to standard chemotherapy. In these situations, alternative forms of first-line therapy may be chosen. As many similar studies are going to be carried out in the near future, correlation of such candidate lists across patients will provide unprecedented information regarding recurrent mutations in specific genes responsible for metastasis and resistance to therapy. In addition, pathway analysis of the mutated genes will allow definition of the functional pathways involved in the above processes.

## Competing interests

The authors declare that they have no competing interests.

## Authors’ contributions

Clinical samples for exome sequencing were provided by LL, LC, AF and WHG. JZ, YS and EL were responsible for exome sequencing data analysis. JZ prepared drafts of the manuscript. WDF and JM supervised data analysis. All authors contributed to all the final manuscript. All authors read and approved the final manuscript.

## Pre-publication history

The pre-publication history for this paper can be accessed here:

http://www.biomedcentral.com/1471-2407/13/146/prepub
